# The Efficacy of a Ketogenic Diet in a Case With Febrile Infection-Related Epilepsy Syndrome in the Chronic Phase: A Case Report

**DOI:** 10.7759/cureus.64149

**Published:** 2024-07-09

**Authors:** Shota Yoneno, Shimpei Baba, Noriko Sumitomo, Kayoko Miyamoto, Kan Takahashi, Yuko Shimizu-Motohashi, Takashi Saito, Hirofumi Komaki

**Affiliations:** 1 Department of Child Neurology, National Center of Neurology and Psychiatry, Tokyo, JPN; 2 Department of Nutrition, National Center of Neurology and Psychiatry, Tokyo, JPN; 3 Department of Pediatrics, Ome Medical Center, Tokyo, JPN; 4 Translational Medical Center, National Center of Neurology and Psychiatry, Tokyo, JPN

**Keywords:** improvement in respiratory function, severe motor and intellectual disabilities, chronic phase, ketogenic diet, febrile infection-related epilepsy syndrome (fires)

## Abstract

Although the treatment strategy for febrile infection-related epilepsy syndrome (FIRES) is improving, current research focuses on acute management. Evidence for the management of the chronic phase is limited. We present the case of a 19-year-old woman with FIRES who showed excellent response to a ketogenic diet (KD) administered in the chronic phase. At the age of four years, she presented with new-onset super-refractory status epilepticus after a febrile episode. She was diagnosed with FIRES and had profound motor and cognitive deterioration and drug-resistant epilepsy. From the age of 17, she experienced numerous seizures that often led to status epilepticus with respiratory failure, necessitating laryngotracheal separation and nocturnal mechanical ventilation. To improve seizure control, we planned a KD for the first time 15 years after the onset of FIRES. We introduced a classic KD (ketogenic ratio, 3:1) using blended meals through gastrotomy. Two months after starting the KD, she experienced a decrease in seizure frequency and duration. Moreover, as unexpected stabilization of respiration was achieved, mechanical ventilation was stopped. Our case implies that KD may be a promising treatment option for patients with FIRES in the chronic phase, as is believed to be the case in the acute phase.

## Introduction

Febrile infection-related epilepsy syndrome (FIRES) is a subgroup of new-onset refractory status epilepticus (SE) that predominantly occurs in children and adolescents. Super-refractory SE (SRSE) develops rapidly within two weeks of the onset of a prior febrile infection. In the chronic phase, most survivors have drug-resistant multifocal epilepsy and varying degrees of intellectual disabilities or learning difficulties [1, 2].

Although the optimal treatment strategy for FIRES has not been established, recent studies have reported the efficacies of several treatment options, such as ketogenic diet (KD), interleukin 1 (IL-1) receptor antagonists, IL-6 antagonists, and intrathecal steroids, for better management of SRSE in the acute phase [3]. Among them, the effectiveness of KD has attracted the attention of clinicians. The recent recommendation described that KD should be administrated as early as possible once FIRES is suspected [4,5]. Conversely, evidence of effective treatments for the chronic phase of FIRES is scarce. As a palliative therapy for drug-resistant epilepsy, the KD should be introduced in patients with chronic FIRES; however, few studies have described the efficacy of the KD at this stage [6].

Here, we present the case of a 19-year-old woman with severe motor and intellectual disabilities (SMID) secondary to FIRES. She showed excellent seizure amelioration and improvement in respiratory function after administration of the KD, which was attempted for the first time 15 years after the onset of FIRES.

## Case presentation

A four-year-old girl who had passed typical developmental milestones presented with new-onset SRSE five days after a febrile episode. She was diagnosed with FIRES and treated with barbiturate coma under mechanical ventilation, intravenous immunoglobulin, and pulsed methylprednisolone. Infectious encephalopathy or metabolic diseases such as mitochondrial disease were less likely based on laboratory findings. There was no family history. About two months after the onset of FIRES, profound motor and cognitive deterioration and drug-resistant epilepsy (DRE) remained as sequelae. She became bedridden, required tube feeding using nasogastric tubes, and could not communicate with others verbally or non-verbally. At the age of six, she was referred to our hospital for further management of epilepsy. However, daily to weekly focal-onset tonic seizures persisted despite the administration of multiple antiseizure medications (ASMs).

**Figure 1:**
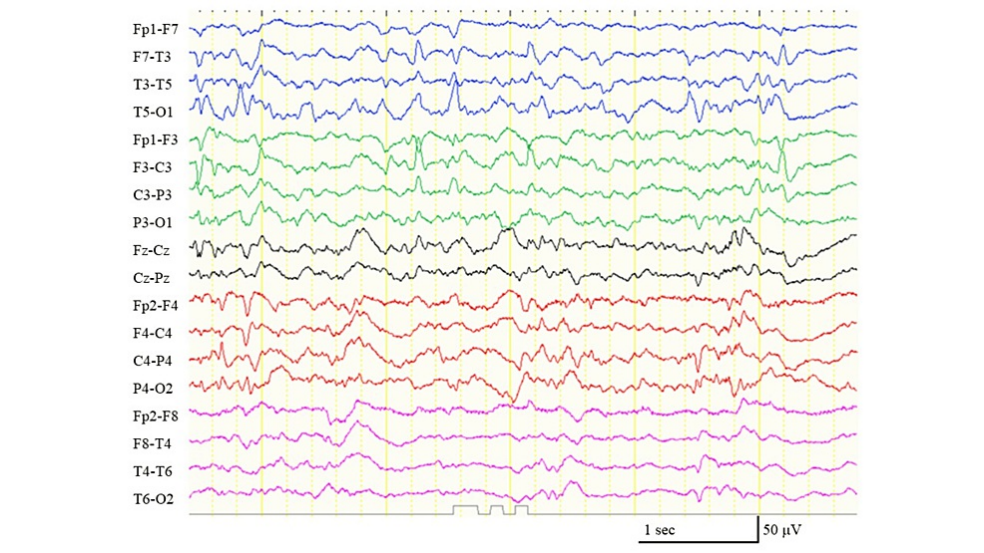
The electroencephalogram before administration of the ketogenic diet. The interictal sleep electroencephalogram shows multifocal epileptiform discharges and a lack of physiological background activities.

At the age of 17 years, her seizures were exacerbated; focal onset tonic seizures, which occurred in each hemisphere, appeared over 20 times a day and were accompanied by severe desaturation, which often resulted in SE. Interictal electroencephalography (EEG) revealed multifocal epileptic discharges (Figure 1) and a lack of physiological background activity.

**Figure 2:**
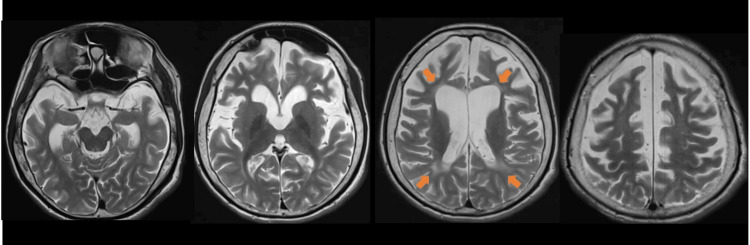
Brain magnetic resonance imaging at the age of 17 years before administration of the ketogenic diet. On axial T2-weighted images, diffuse cerebral atrophy is observed. Diffuse T2 hyperintense white matter lesion is also noted (arrows).

Brain magnetic resonance imaging (MRI) revealed diffuse cerebral atrophy and white matter lesions (Figure 2).

**Figure 3:**
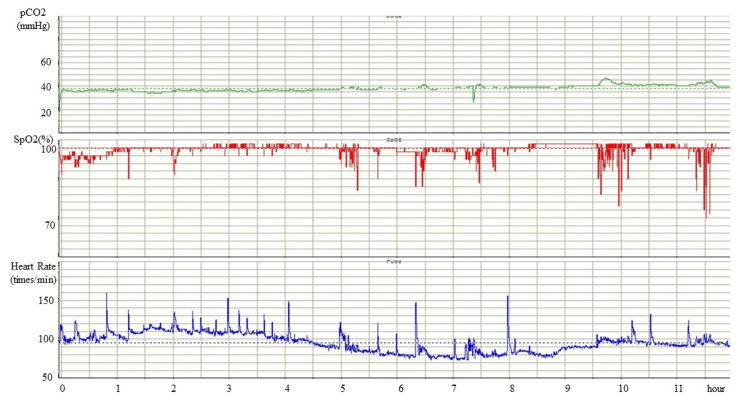
Percutaneous end-tidal CO2 (EtCO2) monitoring before initiating the ketogenic diet. Under mechanical ventilation, percutaneous EtCO_2_ monitoring reveals frequent and severe dips in SpO_2_ accompanied by an elevation in heart rate. These findings are indicative of epileptic seizures.

The maximum doses of zonisamide, lacosamide, carbamazepine, and topiramate did not prevent SE development. Adjunctive high-dose phenobarbital, whose serum concentration was aimed at over 60 μg/ml, was administrated. Under these medications, she was admitted to the emergency hospital every two to three months for SE with/without respiratory failure, aspiration pneumonia, and severe hypothermia. At the age of 18 years, we performed a laryngotracheal separation procedure and gastrostomy sequentially and introduced nocturnal mechanical ventilation to prevent sudden death. However, frequent short SpO_2_ dips were captured by percutaneous end-tidal CO_2_ (EtCO2) monitoring, accompanied by heart rate elevation (Figure 3), which we regarded as habitual seizures. Furthermore, significant SpO_2_ dips persisted for >30 minutes (Figure 3).

**Figure 4:**
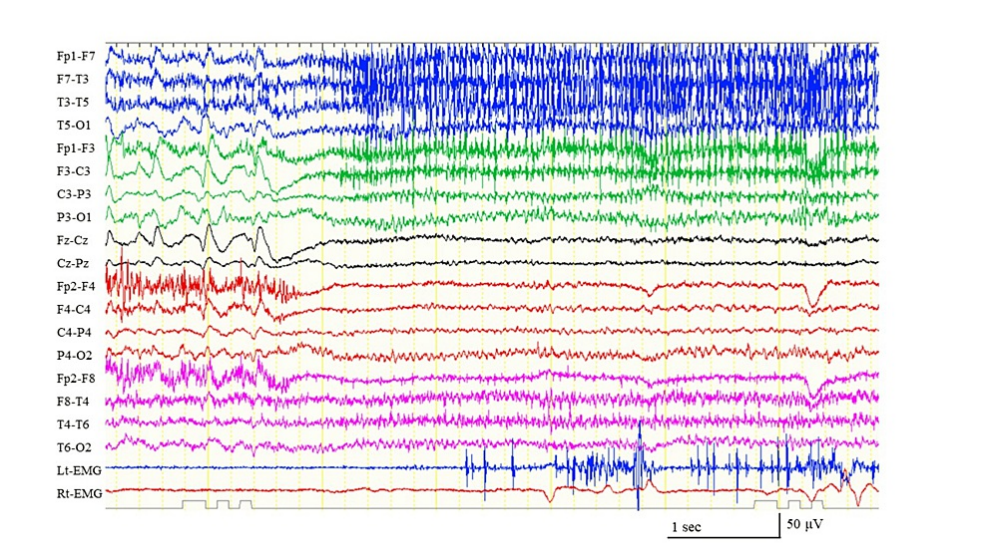
The ictal electroencephalogram before administration of the ketogenic diet. The ictal electroencephalogram (EEG) begins with generalized attenuation and subsequent generalized low-amplitude fast activity. The patient exhibited irregular breathing and left eye deviation. We could not determine the seizure focus based on the ictal EEG findings; however, we determined the seizure to be a focal onset non-motor seizure based on the ictal phenomena.

At that time, she was under appropriate mechanical ventilation, and the ward nurses who were in charge did not notice apparent SE, nor did they need to perform tracheal suctioning to remove the sputum. The video EEG revealed that the patient also had focal onset non-motor seizures accompanied by irregular breathing and left eye deviation (Figure 4).

Therefore, we assumed that the large dips indicated seizure clustering with minor phenomena. At the age of 19 years, we initiated a classic KD using blended meals through gastrotomy without a fasting period. The KD therapy was initiated at a 1:1 ketogenic ratio and gradually advanced to a 3:1 ratio in 11 days (Table 1).

**Table 1:** An example of a ketogenic diet (ketogenic ratio, 3:1). Every dish was processed using the blender and supplied to the patient via a gastrostomy tube.

Meal	Food	Energy (kcal) / nutrition
Breakfast	Fish (mackerel)/45 g, spinach/30 g, Chinese chive/30g, salt/1 g, partially hydrolyzed guar gum/2 g, vegetable oil 17 g, medium-chain triglyceride (MCT) oil 5 g	Energy: 297 kcal, protein: 10.9 g, fat: 27 g, carbohydrate: 0.7 g
Lunch	Pork/45 g, onion/15 g, broccoli/30 g, salt/1 g, ginger 0.5 g, partially hydrolyzed guar gum/2 g, vegetable oil/17 g, MCT oil/5 g	Energy: 314 kcal, protein: 9.6 g, fat: 29.2 g, carbohydrate: 1.7 g
Dinner	Egg/58 g, tofu/30 g, bok choy/40 g, carrot/10 g, salt/1 g, ginger/0.5 g, partially hydrolyzed guar gum/2 g, vegetable oil/17 g, MCT oil/5g	Energy: 309 kcal, protein: 8.5 g, fat: 28.6 g, carbohydrate: 1.6 g

**Figure 5:**
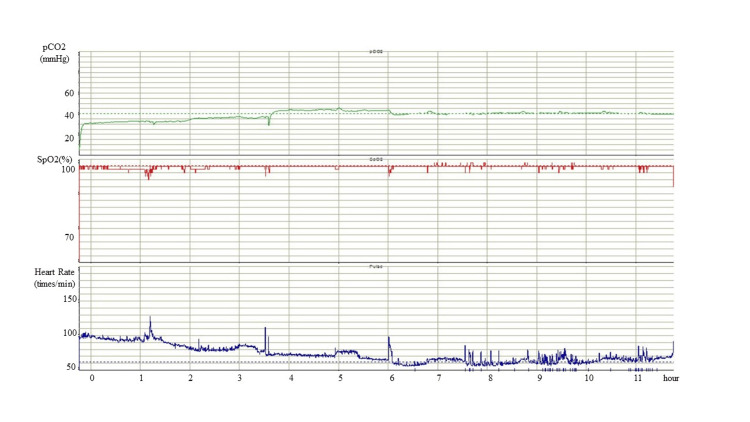
Percutaneous end-tidal CO2 (EtCO2) monitoring after initiating the ketogenic diet. Five months after the administration of the ketogenic diet, the patient showed significant improvements in both the frequency and severity of SpO_2_ dips. At the time this was recorded, mechanical ventilation had been discontinued.

Two weeks after KD initiation, we confirmed the elevation of the serum ketone bodies and positive urinary ketones (Table 2). There were no severe acute complications during the KD, except for hyperuricemia. Two months after starting the KD, her parents noticed the disappearance of clinical seizures. Although her parents voluntarily stopped using the ventilator because of a reduction in clinical seizures, she did not exhibit any apparent desaturating episodes. We assessed her condition five months after starting the KD. Twenty-four hours of video EEG monitoring showed no improvement in the frequency of epileptiform discharges compared with that recorded before the KD. It revealed only four episodes of 10-second-length focal onset non-motor seizures; her parents might not have noticed. However, percutaneous EtCO_2_ monitoring revealed significant improvements in the frequency and severity of SpO_2_ dips and a reduction in heart rate elevation (Figure 5).

**Table 2:** Laboratory findings of serum ketone bodies and urinary ketones. Abbreviations: KD, ketogenic diet

	Total ketone body (μmol/L)	Acetoacetic acid (μmol/L)	3-hydroxybutyric acid (μmol/L)	Urinary ketones
Two weeks after starting KD therapy	5037	1703	3334	3+
At seizure recurrence after starting KD therapy	2714	577	2137	1+

Nine months after starting KD therapy, she was admitted to our hospital with cluster seizures, which was the first emergency admission under the KD therapy. The seizure semiology was the same as before the KD administration. A urine test revealed a decrease in urinary ketones, and a blood test showed a reduction in ketone body levels (Table 2), probably because the KD was insufficiently prepared because of the caregiver’s health issues.

We adequately reintroduced KD therapy, resulting in a prompt decrease in seizure frequency and severity. At the latest follow-up, which was one year and three months after starting the KD, the patient was kept under reasonable seizure control despite reduced phenobarbital dosage and terminated zonisamide administration. No emergency admission was required, except for the aforementioned episode.

## Discussion

Herein, we present a case of FIRES in which a KD was initiated for the first time during the chronic phase. She exhibited remarkable improvement in seizure control and respiratory function, enhancing the quality of life of both the patient and the caregivers. Limited information is available on the efficacy of a KD in patients during the chronic phase. For example, although the recent recommendation stated that KD should be continued if it was influential in the acute phase [4], few studies investigated the efficacy of KD that was administrated in the chronic phase. The KD can be widely administered to patients with DRE. Our case showed that it could ameliorate the frequency and severity of seizures in patients with FIRES in the chronic phase, resulting in unexpected respiratory stabilization. We are of the opinion that our case will contribute to managing the chronic phase of FIRES, as the KD remains a viable treatment option, even when not attempted during the acute phase, regardless of the severity of the patient’s disability.

In addition to being effective in treating refractory epilepsy, a KD has multiple other effects, such as improving cognitive function in conditions, such as Alzheimer's disease and Parkinson's disease [7]. However, to the best of our knowledge, few reports have indicated that a KD improves respiratory function. In the present case, we assumed that the improvement in respiratory function was attributable to a reduction in the seizure burden. She was on high-dose phenobarbital therapy, raising concerns that over-sedation might have contributed to respiratory suppression. However, EtCO_2_ monitoring five months after starting the KD was conducted under the same conditions as ASMs, as it revealed an improvement in SpO2 dips; therefore, we considered oversedation by ASMs to be less likely. We could not determine whether the KD had a beneficial effect on the underlying pathomechanism of FIRES. Several studies that analyzed chronological brain MRIs in patients with FIRES demonstrated progression of brain atrophy despite their stable chronic course, suggesting that the pathogenic process is ongoing even in the chronic stage [8,9]. Further studies are required to elucidate whether the KD is more effective in patients with chronic FIRES than in those with epilepsy of other etiologies.

Although the prognosis of FIRES varies [10], few reports have focused on managing the chronic phase in SMID. Indeed, SMID is not considered a contraindication for KD; we were concerned whether our patient could tolerate its administration as she had severe handicaps in gross motor and respiratory function and episodes of recurrent emergent administration. Consequently, we did not experience difficulty in introducing and maintaining the KD, partially because of the utility of the gastrostomy tube. We hope that this report will encourage those clinicians who hesitate to introduce KD to patients with FIRES because of severe and multiple handicaps.

## Conclusions

We report a case of FIRES in the chronic phase in which the patient experienced a dramatic improvement in seizure control and respiratory function after KD administration. The improvement in respiratory function could have mainly been derived from a reduction in seizure burden. The patient had severe and multiple disabilities, which might make clinicians hesitate to introduce a KD. However, we were able to safely introduce and maintain the KD. We believe that KD is a viable treatment option for patients with FIRES in the chronic phase, even if it is not implemented during the acute phase or if the patients have severe disabilities.
